# Monitoring of immunity against leukemia stem cell in CML patients after cessation of TKI

**DOI:** 10.1186/2051-1426-3-S2-P260

**Published:** 2015-11-04

**Authors:** Maiko Matsushita, Koji Ozawa, Saori Kanchi, Akane Uchiumi, Takuma Suzuki, Daiju Ichikawa, Eri Matsuki, Masatoshi Sakurai, Daiki Karigane, Hidenori Kasahara, Hideaki Nakajima, Shinichiro Okamoto, Yutaka Hattori

**Affiliations:** 1 Faculty of Pharmacy, Keio University, Tokyo, Japan; 2 Division of Pharmacy, National Cancer Center Hospital, Tokyo, Japan; 3 Division of Hematology, Keio University, Tokyo, Japan

## Purpose

Tyrosine kinase inhibitors (TKIs) have improved overall survival of patients with chronic myeloid leukemia (CML). Moreover, it has been reported that some of the patients achieve treatment-free remission (TFR) after cessation of TKIs, even though leukemic stem cells (LSC) of CML are considered to be resistant to TKIs. Therefore, these findings suggest that remaining LSCs could be eradicated by immune system after withdrawal of TKIs. In order to clarify the role of anti-LSC immunity in achieving TFR, we tried to detect cytotoxic T cells against one of the LSC antigens, KU-MEL9 in CML patients.

## Methods

Fresh blood samples were obtained from 16 CML patients who stopped imatinib after maintained complete molecular response (CMR) for more than two years at 0,3, and 6 months after cessation of imatinib. Mononuclear cells were stimulated with LSC antigen, KU-MEL9-derived nanomer peptide twice *in vitro*, then stained with anti-KU-MEL9-specific dextramer and analyzed by flow cytometry. Immunophenotype of lymphocytes was also determined by flow cytometry.

## Results

We detected KU-MEL9-specific CTLs in 3 of 16 patients (Table 1). All of these patients remained in CMR at least 12 months after cessation of imatinib. On the other hand, none of 4 patients who showed molecular relapse presented with KU-MEL9-specific CTLs. The relapse rate of patients without KU-MEL9-CTLs was 31.3%, as compared to 0 % in KU-MEL9-CTL-positive patients. Percentage of CD4^+^ CD25^+^ cells was higher in patients who experienced loss of CMR.

**Table 1:** 

Patients	0m	3m	6m	Clinical course
1	-	-	-	Molecular relapse
2	-	-	-	CMR
3	-	-	+	CMR
4	-	-	-	CMR
5	-	-	-	CMR
6	-	+	-	CMR
7	-	-	-	CMR
8	-	-	-	Molecular relapse
9	-	-	-	CMR
10	-	-	-	CMR
11	-	-	-	Molecular relapse
12	-	-	-	Molecular relapse
13	-	-	-	CMR
14	+	-	-	CMR
15	-	-	-	CMR
16	-	-	-	CMR

## Conclusion

We found LSC antigen-specific T cells only in patients who achieved TFR. Although the patient size is small, these data suggest that anti-LSC CTLs may play a role in eradicating remaining LSCs after imatinib treatment and LSC antigen-specific immunotherapy could be useful to increase TFR rate.

## Consent

Written informed consent was obtained from the patients for publication of this abstract and any accompanying images. A copy of the written consent is available for review by the Editor of this journal.

